# Estimativas do Risco Cardiovascular em Dez Anos na População Brasileira: Um Estudo de Base Populacional

**DOI:** 10.36660/abc.20190861

**Published:** 2021-03-03

**Authors:** Deborah Carvalho Malta, Pedro Cisalpino Pinheiro, Renato Azeredo Teixeira, Isis Eloah Machado, Filipe Malta dos Santos, Antônio Luiz Pinho Ribeiro

**Affiliations:** 1 Universidade Federal de Minas Gerais Belo HorizonteMG Brasil Universidade Federal de Minas Gerais, Belo Horizonte, MG - Brasil.; 2 Universidade Federal de Ouro Preto Ouro PretoMG Brasil Universidade Federal de Ouro Preto, Ouro Preto, MG - Brasil.

**Keywords:** Doenças Cardiovasculares, Fatores de Risco, Colesterol, Diabetes Mellitus, Hipertensão, Epidemiologia

## Abstract

**Fundamento:**

As doenças cardiovasculares são a principal causa de morbimortalidade, altos custos com saúde e perdas econômicas importantes. O escore de Framingham tem sido amplamente utilizado para estratificar o risco dos indivíduos avaliados, identificando aqueles com risco maior para que sejam implementadas medidas de prevenção direcionadas para esse grupo.

**Objetivos:**

Estimar o risco cardiovascular em 10 anos da população brasileira adulta.

**Métodos:**

Estudo transversal, utilizando dados laboratoriais de uma subamostra da Pesquisa Nacional de Saúde. Para calcular o risco cardiovascular, utilizou-se o escore de Framingham, estratificado por sexo.

**Resultados:**

A maioria das mulheres (58,4%) apresentou baixo risco cardiovascular, 32,9%, risco médio e 8,7%, risco elevado. Entre homens, 36,5% apresentaram risco cardiovascular baixo, 41,9%, risco médio e 21,6%, risco elevado. O risco aumentou com a idade e foi elevado na população com baixa escolaridade. A proporção dos componentes do modelo de Framingham, por grupos de risco e sexo, mostra que, no risco elevado entre mulheres, os indicadores que mais contribuíram para o risco cardiovascular foram: a pressão arterial sistólica, colesterol total, HDL, diabetes e tabagismo. Entre homens, pressão arterial sistólica, colesterol total, HDL, tabagismo e diabetes.

**Conclusões:**

Trata-se do primeiro estudo nacional com dados laboratoriais a estimar o risco de doença cardiovascular em dez anos. Os escores de risco são úteis para subsidiar as práticas de prevenção dessas doenças, considerando o contexto clínico e epidemiológico.

## Introdução

As doenças cardiovasculares (DCV) foram responsáveis por cerca de 17,9 milhões de mortes em 2016, cerca de 31% das mortes globais, constituindo as causas mais frequentes de morbimortalidade.^1^^–^^3^ Também no Brasil, em 2016, as DCV lideraram as maiores taxas de mortalidade e anos de vida perdidos ajustados por incapacidade, em ambos os sexos.^4^^,^^5^ As DCV destacam-se, ainda, pelos maiores custos com internações e tratamento no Sistema Único de Saúde (SUS), além de custos indiretos ocasionados pela redução da produtividade, afastamento do trabalho e os efeitos negativos sobre a qualidade de vida das pessoas afetadas e familiares.^6^

Os estudos de coorte *Framingham Heart Study*, iniciados em 1948, foram pioneiros na identificação da associação entre os principais fatores de risco (hipertensão arterial, colesterol elevado e tabagismo), com a doença coronariana.^7^ Na sequência desses achados, surgiram diretrizes e protocolos concentrados em um único fator de risco como a hipertensão arterial^8^ ou colesterol,^9^ para prevenção das DCV. Estudos da Nova Zelândia foram pioneiros, em 1993, na utilização de fatores de risco múltiplos na previsão de risco cardiovascular.^10^ Oriundos da equipe de Framingham, os estudos propuseram uma sistematização por sexo e faixa etária, que previa o risco de desenvolvimento de doença coronariana na próxima década, mediante escores calculados a partir dos valores da pressão arterial sistólica, do colesterol total, da fração HDL do colesterol, do diagnóstico de diabetes e do hábito de fumar.^10^^,^^11^

A proposição de algoritmos de Framingham para previsão de DCV foi incorporada ao Terceiro Relatório do Painel de Especialistas em Detecção, Avaliação e Tratamento do colesterol alto no sangue no Painel III de Tratamento em adultos, em 2001.^12^ Houve validação desses algoritmos em brancos e pretos nos Estados Unidos,^13^^,^^14^ em diversas populações da Europa, na região do Mediterrâneo, Ásia, e em todo o mundo, com bom desempenho.^15^^–^^19^

Outras adaptações se seguiram, com destaque para o Risco Cardiovascular Geral, em 2008, proposto pelo grupo de Framingham,^20^ que busca estimar o risco de eventos cardiovasculares em 10 anos como: doença arterial coronariana (DAC), acidente vascular cerebral (AVC), doença arterial obstrutiva periférica ou insuficiência cardíaca.^20^ Esse escore tem sido muito utilizado no mundo e também foi empregado no país pelas diretrizes brasileiras para conhecer e estimar o risco cardiovascular (CV) absoluto em dez anos.^21^ Esses escores permitem ações preventivas, principalmente por dirigir a estratégia populacional para a busca e identificação da população de alto risco, buscando oportunidades para a sua prevenção.^22^

Visando conhecer o perfil de saúde da população brasileira, o Ministério da Saúde e o Instituto Brasileiro de Geografia e Estatística (IBGE) realizaram a Pesquisa Nacional de Saúde (PNS), um amplo inquérito domiciliar que reuniu informações de abrangência nacional sobre a população, incluindo em seu questionário informações sobre fatores de risco de DCV e, nos anos de 2014 e 2015, realizou-se a coleta de exames laboratoriais, o que possibilita avançar em análises de RCV representativas da população brasileira, na medida em que estimativas anteriores baseavam-se em estudos de populações especificas como estudos hospitalares^23^ ou estudos de coorte resultantes de populações de servidores de universidades brasileiras.^24^

Assim, o presente estudo teve o objetivo estimar o risco cardiovascular em dez anos da população brasileira adulta, segundo dados laboratoriais da PNS.

## Métodos

Trata-se de estudo transversal realizado a partir de dados secundários da Pesquisa Nacional de Saúde (PNS). A PNS é uma pesquisa domiciliar integrante do Sistema Integrado de Pesquisas Domiciliares (SIPD), do Instituto Brasileiro de Geografia e Estatística (IBGE).^25^^,^^26^ O componente laboratorial foi coletado em 2014 e 2015 e as metodologias do processo amostral da PNS e da subamostra do laboratório encontram-se detalhadas em estudos anteriores.^25^^,^^27^^,^^28^ A subamostra laboratorial obtida foi de 8.952 pessoas e, visando a correção de possíveis vieses nas análises estatísticas, foram utilizados pesos de pós-estratificação segundo sexo, idade, escolaridade e região.^28^^,^^29^ O procedimento de ponderação utilizou variáveis disponíveis na amostra e na população de referência, obtidas de fontes externas, segundo dados do Censo 2010 do IBGE, para ajustar a distribuição da amostra coletada na pesquisa domiciliar com aquela verificada para o conjunto completo da população brasileira. A escolha das variáveis utilizadas na construção dos pesos levou em consideração as características da população excluída, de forma a minimizar o vício de representação. Desta forma, utilizando os pesos de pós-estratificação, a amostra do laboratório torna-se representativa da população adulta brasileira.^28^^,^^29^

O sangue coletado no laboratório foi centrifugado, e as amostras de soro e plasma, armazenadas em um refrigerador a 4 ºC, foram analisadas por equipamento automatizado (COBAS MIRA PLUS, Roche) regularmente calibrado. Dentre os exames coletados, a hemoglobina glicada (HbA1c) foi colhida em tubo com ácido etilenodiamino tetra-acético (EDTA) e dosada por cromatografia líquida de alta performance por troca iônica (HPLC — High Pressure Liquid Chromatography). Utilizou-se o ponto de corte da Organização Mundial de Saúde (OMS), e a American Diabetes Association recomenda o valor de HbA1c ≥ 6,5% para o diagnóstico de diabetes mellitus (DM).^29^ O colesterol total (CT) e a lipoproteína de alta densidade (HDL — High-Density Lipoprotein) foram colhidos em tubo gel e calculados os valores para a população brasileira.^30^

A pressão arterial foi mensurada após a explicação do procedimento ao paciente, que: deveria repousar ao menos cinco minutos em ambiente calmo; não estar com a bexiga cheia; não ter praticado exercícios físicos 60 a 90 minutos antes; não ter ingerido bebidas alcoólicas, café ou alimentos; não ter fumado 30 minutos antes; manter pernas descruzadas, pés apoiados no chão, dorso recostado na cadeira, relaxar e não falar durante a aferição.^31^ Ao todo, foram feitas três aferições com intervalos de dois minutos entre elas, usando um esfigmomanômetro de coluna de mercúrio devidamente calibrado. Ao final, a média das três leituras foi registrada como valor definitivo para a análise dos dados.

O tabagismo foi avaliado a partir das seguintes perguntas: “Você é ou já foi fumante, ou seja, já fumou, ao longo da vida, pelo menos 100 cigarros?”; e “Quantos cigarros, atualmente, você fuma por dia?”.

A pontuação para estimar o RCV geral seguiu a proposta de Framingham^20^ e considera sexo, idade, medida do colesterol total, colesterol HDL, medidas obtidas quando à pressão arterial tratada e não tratada, fumante (sim ou não), diabetes (sim ou não). Foram realizados cálculos distintos para homens e mulheres, sendo calculados riscos específicos por sexo, idade e considerados os fatores de risco (FR) descritos abaixo.^20^ Foram excluídos da análise indivíduos com idade inferior a 30 e superior a 74, mantendo os mesmos grupos etários da coorte utilizada na estimativa do risco.^20^ Da mesma forma, foram excluídos da análise indivíduos que declararam ter sido diagnosticados por um médico com doença do coração ou acidente vascular cerebral (AVC).

As pontuações consideraram a proposta de D’Agostino et al.,^20^ detalhada em outra publicação,^20^ e que foi adotada no Brasil, em 2013, pela Sociedade Brasileira de Cardiologia, denominada Escore de Risco Global (ERG).^21^ A idade foi autorreferida pelo participante e considerou faixas de idade de 30 a 34, 35–39, 40–44, 45–49, 50–54, 55–59, 60–64, 65–69, 70–74, 75 anos ou mais. A pontuação entre homens varia de 0 a 15 pontos e entre mulheres, varia de 0 a 12 pontos.

A pontuação dos fumantes do sexo masculino foi de 4 pontos e do sexo feminino foi de 3 pontos. A pressão arterial (PA) atribui pontuação de modo diferenciado entre aqueles em tratamento e que não estavam em tratamento medicamentoso, considerando a pergunta: “Você usou remédio para pressão alta nos últimos 15 dias?”. A pontuação entre homens variou de -2 a 3 (em tratamento) e de 0 a 4 (sem tratamento); entre mulheres, de -1 a 7 (em tratamento) e de -3 a 5 (sem tratamento).^20^

Em relação aos exames laboratoriais, os pontos de corte e a pontuação foram:

- Diabetes: usou-se a medida da Hemoglobina Glicada (HbA1c <6,5% = 0 para ambos os sexos; HbA1c ≥ 6,5% para homens = 3 pontos; para mulheres = 4 pontos), ou o diagnóstico da doença por um médico.
- Colesterol total (CT) para mulheres: CT<160 mg/dl = 0 pontos, CT 60–199 mg/dl = 1 ponto, CT≥200 -239 mg/dl = 3 pontos; CT ≥240 -279 mg/dl = 4 pontos, CT≥280 = 5 pontos. Para homens: CT<160 mg/dl = 0 pontos, CT 160–199 mg/dl = 1 ponto, CT≥200 -239 mg/dl = 2 pontos; CT≥240 -279 mg/dl = 3 pontos, CT≥280 = 4 pontos.
- Colesterol HDL para homens: ≥60 mg/dL= -2 pontos, HDL 50–59 = -1 ponto, HDL 45–49= 0 ponto, 35–44= 1, <35 mg/dL = 2 pontos. Para mulheres: ≥60 mg/dL= -2 pontos, HDL 50–59 mg/dL = -1 ponto, HDL 45–49 = 0 ponto, 35–44=1, <35 mg/dL = 2 pontos.

O estudo estimou o ERG geral para homens e mulheres e os respectivos intervalos de confiança (IC95%). As análises foram realizadas no Stata, versão 13. Conforme as diretrizes da Sociedade Brasileira de Cardiologia, utilizou-se os seguintes pontos de corte do risco cardiovascular em 10 anos: a) baixo RCV<5%, médio RCV (5 a <20%) e alto RCV (≥20%).^21^^,^^32^

O questionário da PNS e as variáveis já foram divulgados em publicações anteriores e maiores detalhes podem ser vistos em outras publicações.^27^ Conforme previsto no protocolo da pesquisa, todos os resultados de exames foram informados ao usuário pelo laboratório responsável e, em casos de resultados alterados, os usuários foram orientados a procurar assistência médica na rede pública e, em casos de risco extremo, os usuários foram contatados diretamente pelo laboratório conveniado ou pelo Ministério da Saúde, visando o atendimento imediato.^28^

Ressalta-se ainda que a PNS foi aprovada pela Comissão Nacional de Ética em Pesquisa sob o nº 328.159, de 26 de junho de 2013. Todos os indivíduos foram consultados, esclarecidos, e aceitaram participar da pesquisa.

## Resultados

O estudo mostra que, em mulheres, 58,4% apresentaram risco cardiovascular baixo, (<5%); 32,9%, ERG médio (5 a 19%) e 8,7%, ERG elevado (>=20%). O ERG elevado em mulheres aumentou com a idade, passando de 0,1% entre 40 e 44 anos para 9,3% entre 50 e 54 anos, 10,6% entre 55 e 59 anos, 29% entre 60 e 64 anos, 29,9% entre 65 e 69 anos e 38,4% entre 70 e 74 anos. A diferença no ERG segundo anos de escolaridade foi cerca de cinco vezes entre a escolaridade elevada (12 anos e mais de estudo) e <8 anos de estudo (3,2%: IC95% 2,4–4,4 versus 15,7%: IC95% 13,5–18,3). Quem tem plano de saúde teve menor ERG, 5,4% (IC95% 3,9–7,3) versus 10,2% (IC95% 8,8–11,8) do que aqueles que não têm. As mulheres de cor preta apresentaram maior proporção no grupo de risco mais elevado (>=20%), 14,4% (IC95% 9,7–20,9), do que aquelas de cor branca, 7,3 (IC95% 5,8–9,1). A autoavaliação de saúde ruim mostrou o maior diferencial entre mulheres e apresentou gradiente, sendo os extremos: mulheres que se autoavaliam com saúde muito boa 2,9% (IC95% 1,3–3,6) e saúde muito ruim, 25,6% (12,7–45,0) (Tabela 1).

**Tabela 1:** Distribuição proporcional das variáveis selecionadas por grupos de risco cardiovascular, mulheres, PNS 2013

Variável		Menor 10%		Entre 11% e 20%		Maior igual 20%	
		n	%	n	%	n	%
**Mulheres**		2092	58,4 (56,3; 60,5)	1180	32,9 (31; 35)	312	8,7 (7,6; 9,9)
**Idade**							
	30–34	564	100	0	-	0	-
	35–39	482	94,1 (90,2; 96,5)	30	5.8 (3.4; 9.8)	0	0 (0; 0,3)
	40–44	404	84,9 (80,2; 88,6)	72	15 (11,3; 19,7)	1	0,1 (0; 0,9)
	45–49	353	70,3 (64,8; 75,3)	147	29,3 (24,3; 34,8)	2	0,4 (0,1; 1,6)
	50–54	161	39,2 (33,3; 45,5)	211	51,4 (45,2; 57,6)	38	9,3 (6,3; 13,7)
	55–59	87	22,1 (17,1; 28)	265	67,3 (61; 73)	42	10,6 (7,6; 14,7)
	60–64	28	9,3 (6,2; 13,6)	186	61,7 (54,8; 68,2)	87	29 (23; 35,9)
	65–69	12	4,5 (2,4; 8,5)	172	65,5 (58,2; 72,2)	79	29,9 (23,6; 37,2)
	70–74	2	1,5 (0,5; 4,1)	98	60,2 (50,5; 69,1)	63	38,4 (29,5; 48,1)
**Escolaridade**							
	0–8 anos	572	40,2 (37,1; 43,3)	628	44,1 (41; 47,2)	224	15,7 (13,5; 18,3)
	9 a 11	293	61,5 (55,3; 67,3)	151	31,6 (26,1; 37,7)	33	6,9 (4,5; 10,4)
	12 e mais	1227	72,9 (70; 75,7)	401	23,8 (21,2; 26,7)	54	3,2 (2,4; 4,4)
**Cor**							
	Branca	1003	58,3 (55; 61,6)	591	34,4 (31,3; 37,6)	125	7,3 (5,8; 9,1)
	Preta	169	49,8 (42,7; 56,9)	122	35,8 (29,4; 42,7)	49	14,4 (9,7; 20,9)
	Parda	891	60,1 (57,2; 62,8)	457	30,8 (28,3; 33,5)	135	9,1 (7,6; 10,9)
	Outras	30	69,4 (49,1; 84,2)	11	24,8 (11,4; 45,7)	2	5,9 (2,2; 14,8)
**Região**							
	Norte	150	61,8 (58,5; 65)	63	29,5 (26,5; 32,6)	12	8,7 (7; 10,8)
	Nordeste	561	54,8 (52,1; 57,4)	278	32,7 (30,3; 35,2)	85	12,6 (11; 14,4)
	Sudeste	910	49,9 (46,2; 53,5)	578	36,3 (32,9; 39,9)	152	13,8 (11,6; 16,4)
	Sul	308	50,7 (46,3; 55)	177	36,1 (32,1; 40,2)	46	13,3 (10,7; 16,3)
	Centro-Oeste	164	54,9 (50,3; 59,5)	84	33,1 (28,9; 37,5)	16	12 (9,4; 15,2)
**Plano de saúde**							0(0;0)
	Não	1375	56 (53,5; 58,4)	830	33,8 (31,5; 36,2)	251	10,2 (8,8; 11,8)
	Sim	717	63,6 (59,6; 67,4)	350	31,1 (27,4; 35)	60	5,4 (3,9; 7,3)
**Autoavaliação**							
	Muito boa	313	73,4(67;78,9)	102	23,8(18,5;30)	12	2,9(1,3;6)
	Boa	1188	67,3 (64,3; 70,1)	500	28,3 (25,6; 31,2)	78	4,4 (3,3; 5,8)
	Regular	504	44,3 (40,9; 47,8)	469	41,2 (37,7; 44,8)	165	14,5 (12,1; 17,2)
	Ruim	69	33,1 (26; 41,2)	94	45,1 (37,4; 53,1)	45	21,7 (15,9; 29)
	Muito ruim	17	39,2 (24,5; 56,2)	16	35,1 (22,6; 50,2)	11	25,6 (12,7; 45)

Fonte: Pesquisa Nacional de Saúde, 2013.

Entre homens, 36,5% apresentaram risco cardiovascular baixo (<5%); 41,9%, ERG médio (5 a 19%) e 21,6%, ERG elevado (≥ 20%). O ERG elevado em homens aumentou com a idade, passando de 1,0% entre 40 e 44 anos; 4,9% entre 45 e 49 anos; 17,1% entre 50 e 54 anos, 44,7% entre 55 e 59 anos; 61,5% entre 60 e 64 anos; 78,2% entre 65 e 69 anos; 91,9% após 70 a 74 anos. A diferença no RCV segundo escolaridade foi de cerca de duas vezes, 13,8% (12 anos ou mais de estudo) e 29,8% (<8 anos de estudo). Não foram identificadas diferenças no ERG segundo raça e cor e posse de planos de saúde. A autoavaliação de saúde em homens também apresentou gradiente: muito boa 11,4% (IC95% 8–15,9) e autoavaliação de saúde ruim 39,1% (IC95% 28,8–50,4) (Tabela 2).

**Tabela 2:** Distribuição proporcional das variáveis selecionadas por grupos de risco cardiovascular, homens, PNS 2013

Variável		Menor 10%		Entre 11% e 20%		Maior igual 20%	
		n	%	n	%	n	%
**Homens**		950	36,5 (34,1; 39,1)	1088	41,9 (39,4; 44,4)	562	21,6 (19,7; 23,6)
**Idade**							
	30–34	390	96,4 (92,9; 98,2)	15	3,6 (1,8; 7,1)	0	-
	35–39	342	84,5 (78,9; 88,7)	63	15,5 (11,3; 21,1)	0	-
	40–44	139	38,2 (31,8; 45)	221	60,8 (53,9; 67,2)	4	1 (0,4; 2,4)
	45–49	63	18,7 (13,9; 24,6)	258	76,4 (70,2; 81,7)	16	4,9 (2,7; 8,5)
	50–54	17	5,1 (3; 8,6)	250	77,7 (71,7; 82,7)	55	17,1 (12,7; 22,8)
	55–59	0	-	142	55,3 (47,4; 62,9)	115	44,7 (37,1; 52,6)
	60–64	0	-	99	38,5 (31,3; 46,2)	158	61,5 (53,8; 68,7)
	65–69	0	-	33	21,8 (15,7; 29,4)	118	78,2 (70,6; 84,3)
	70–74	0	-	8	8,1 (4,2; 15,2)	95	91,9 (84,8; 95,8)
**Escolaridade**							
	0–8 anos	237	21,8 (19,1; 24,8)	525	48,4 (44,8; 51,9)	324	29,8 (26,8; 33,1)
	9 a 11	163	41,8 (35,1; 48,8)	145	37,1 (30,7; 44)	82	21,1 (16,2; 27)
	12 e mais	550	48,9 (44,8; 53,1)	418	37,2 (33,3; 41,3)	155	13,8 (11,4; 16,7)
**Cor**							
	Branca	408	33,5 (29,8; 37,5)	532	43,7 (39,8; 47,7)	277	22,8 (19,8; 26,1)
	Preta	92	34 (26,8; 42,1)	129	47,9 (39,6; 56,3)	49	18,1 (12,7; 25)
	Parda	441	40,5 (36,8; 44,2)	415	38,2 (34,8; 41,6)	233	21,4 (18,8; 24,3)
	Outras	10	41,1 (23,1; 61,9)	12	47,4 (27; 68,7)	3	11,5 (4,9; 24,7)
**Região**							
	Norte	70	39,7 (35,7; 43,7)	78	44,3 (40,3; 48,3)	28	16,1 (13,4; 19,2)
	Nordeste	274	40,2 (36,9; 43,6)	283	41,6 (38,4; 45)	124	18,2 (15,7; 20,9)
	Sudeste	391	34,2 (29,6; 39,2)	463	40,5 (35,8; 45,4)	288	25,2 (21,6; 29,3)
	Sul	143	35,9 (30,3; 42)	170	42,9 (37,2; 48,8)	84	21,2 (17,1; 25,9)
	Centro-Oeste	72	35,8 (30; 41,9)	93	45,9 (40,1; 51,8)	37	18,3 (14,4; 23)
**Plano de saúde**							
	Não	649	34,9 (32,1; 37,8)	790	42,6 (39,7; 45,4)	418	22,5 (20,3; 24,9)
	Sim	302	40,6 (35,5; 45,9)	298	40,1 (35,1; 45,3)	143	19,3 (15,7; 23,5)
**Autoavaliação**							
	Muito boa	210	52,8 (45,7; 59,8)	143	35,8 (29,5; 42,7)	45	11,4 (8; 15,9)
	Boa	566	41 (37,5; 44,6)	562	40,7 (37,3; 44,2)	254	18,4 (15,9; 21,1)
	Regular	159	22,7 (19,2; 26,7)	323	46,2 (41,6; 50,8)	218	31,1 (27,1; 35,4)
	Ruim	13	13,3 (8,4; 20,5)	46	47,6 (36,3; 59,2)	37	39,1 (28,8; 50,4)
	Muito ruim	2	7,6 (2,2; 22,7)	14	61,3 (39,1; 79,7)	7	31,1 (14,7; 54,2)

Fonte: Pesquisa Nacional de Saúde, 2013.

**Figura 1:**
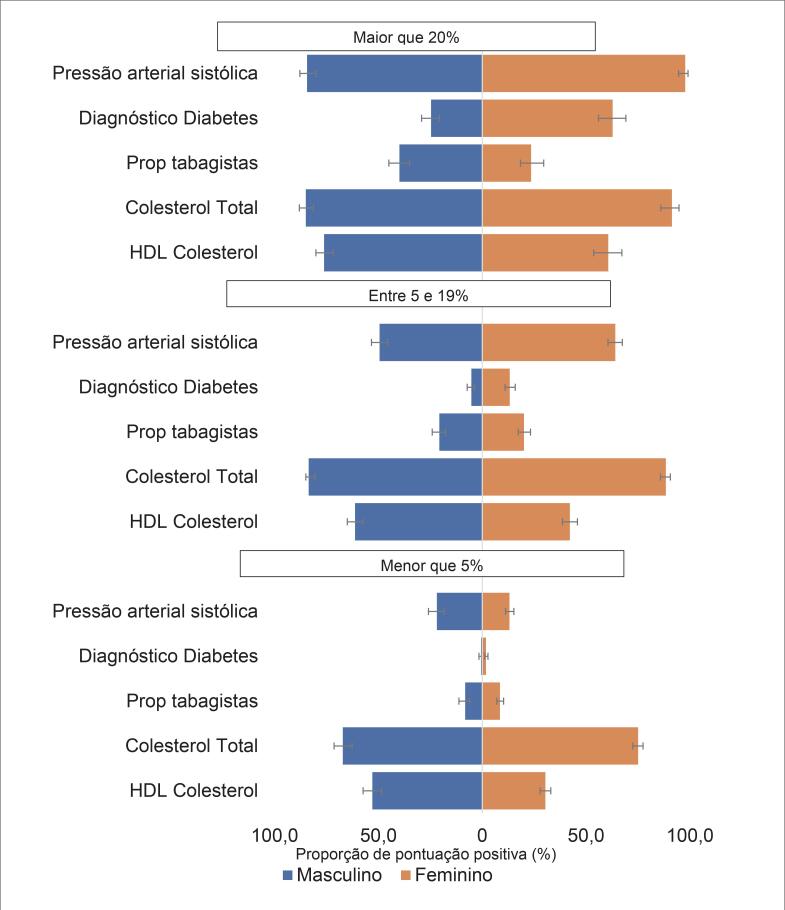
Distribuição proporcional dos componentes do modelo de Framingham por grupos de risco alto, médio, baixo, respectivamente, por sexo, PNS, 2013.

A Figura 1 mostra a distribuição proporcional dos componentes do modelo de Framingham por grupos de risco, que contribuíram positivamente (maior que zero) para a pontuação total atribuída. No risco elevado entre mulheres, os indicadores que mais contribuíram para o ERG foram: pressão arterial sistólica (97,7%), colesterol total (91,3%), diabetes (62,8%), HDL (60,6%) e, por último, o tabagismo. Entre homens: colesterol total (85%), pressão arterial sistólica (84,3%), HDL (76,2%), tabagismo (39,9%) e diabetes (24,7%).

Fonte: Pesquisa Nacional de Saúde, 2013.

## Discussão

Este constitui o primeiro estudo nacional de base populacional a estimar o ERG para a população adulta brasileira utilizando dados laboratoriais. Para o cálculo, foram empregados algoritmos propostos por D’Agostino et al.,^20^ a partir do estudo de Framingham. Esses modelos foram estimados por meio de funções matemáticas para estimar o risco absoluto de DCV em 10 anos.^20^ O ERG elevado (>=20%) foi encontrado em cerca de 8,7% das mulheres e em cerca de um quinto dos homens. O ERG aumentou com a idade, atingindo cerca de 40% das mulheres entre 70 e 74 anos e quase a totalidade dos homens nessa faixa. O risco quadriplicou entre mulheres de baixa escolaridade e dobrou entre homens. Cabe salientar que há maior concentração de indivíduos com ERG maior que 20% nos grupos menos escolarizados e mais velhos. É provável que parte dessa concentração seja um efeito de coorte, uma vez que, em média, os indivíduos mais velhos são menos escolarizados que os grupos mais jovens.^33^ Nesse sentido, uma parcela importante da concentração de indivíduos menos escolarizados no grupo de risco mais elevado pode ser explicada pela idade mais avançada do grupo e vice-versa. Outras análises, que fogem do escopo do presente trabalho, podem separar os efeitos.

Em relação às brancas, apenas as mulheres negras apresentaram maior proporção no grupo de maior risco. Surpreende a não identificação de diferença (estatisticamente significativa) por raça/cor do percentual de homens com ERG elevado. É provável que parte do potencial diferencial por raça tenha sido captado por outras variáveis correlacionadas como, por exemplo, a idade e a escolaridade. Na amostra, a população masculina de cor branca, comparada a pretos e pardos, apresenta maior concentração relativa em idades mais velhas. Entre mulheres, foi mais elevado o risco entre as que não têm plano de saúde e observou-se gradiente dose-resposta entre RCV e autoavaliação de saúde, chegando a ser mais de oito vezes mais elevado entre a avaliação muito boa e muito ruim e, entre homens, essa diferença foi de cerca de 3 vezes. Os fatores que mais frequentemente contribuíram com o ERG elevado foram a idade, pressão arterial e colesterol elevado.

Várias calculadoras de avaliação de risco foram desenvolvidas para a estimativa do risco de cardiovascular a partir dos estudos de Framingham. O escore atual foi revisado em 2008^20^ e incluiu parâmetros clínicos cardiovasculares adicionais e, embora esse modelo de risco forneça uma estimativa melhorada das DCV, ele ainda sofre com alguns desafios e subestima o risco em mulheres.^21^ A classificação empregada neste estudo utiliza modelos de Cox e covariáveis como idade, colesterol total, colesterol HDL, pressão arterial sistólica tratada e não tratada, uso de medicamento anti-hipertensivo, tabagismo atual e status de diabetes para o cálculo do RCV.^20^ Os autores transformaram as variáveis contínuas em logarítmicas para melhorar a discriminação e calibração dos modelos e para minimizar a influência de observações extremas.^20^

Esses algoritmos foram recomendados pela Sociedade Brasileira de Cardiologia na I Diretriz Brasileira de Prevenção Cardiovascular^21^ e agregam vantagens na identificação de ERG, selecionando indivíduos prioritários para intervenção, com múltiplos fatores de risco, evitando identificar desnecessariamente pessoas com apenas um risco isolado.^20^ Essas estimativas da DCV global apoiam a identificação de pacientes eleitos para medidas de prevenção e tratamento, tornando-se medidas custo-efetivas,^21^ sendo útil para aplicação na atenção primária.

O algoritmo proposto classifica segundo sexo, aumentando a pontuação de risco com o aumento da idade, para o hábito de fumar, pressão arterial (PA) não tratada e diabetes.^20^ Entre mulheres, o algoritmo aumenta nas faixas etárias pós-menopausa e eleva o risco para fatores como fumo e diabetes. Mesmo empregando pontuações mais elevadas para as mulheres, o ERG foi duas vezes mais elevado entre homens.

No Brasil, alguns estudos mediram o RCV entre adultos e idosos, empregando a calculadora de Framingham^34^ como a coorte de Bambuí. Foram avaliados (n=547 entre 30–59 anos) e toda a população idosa (n=1165, 60–74 anos), com RCV entre idosos de 56% entre homens e 21% entre mulheres.^35^ Outro estudo nacional, que avaliou cerca de 15.000 indivíduos atendidos no serviço de check-up do Centro de Medicina Preventiva do Hospital Israelita Albert Einstein entre 2009 e 2015, também identificou proporções semelhantes. O RCV elevado em mulheres foi de 12,3% e entre homens foi de 40,1%.^23^

O risco cardiovascular mais elevado entre homens reflete a presença de estilos de vida menos saudáveis como tabagismo, alimentação inadequada, consumo de álcool, baixa procura de serviços de saúde, não uso de medicamentos, o que já foi documentado em diversos outros estudos nacionais.^35^^–^^37^

O aumento do risco com a idade tem sido atribuído ao envelhecimento, aumento da PA, que pode afetar cerca de 60% dos idosos, segundo dados da PNS.^31^ As explicações seriam as alterações inerentes ao envelhecimento, com maior enrijecimento das artérias, maior resistência vascular periférica e comorbidades entre os idosos^21^^,^^38^^–^^40^

No caso das mulheres, a elevação do ERG na faixa etária após a menopausa decorre da perda do efeito protetor hormonal nessa época da vida. O aumento da hipertensão em mulheres também tem sido descrito pelo crescimento da obesidade central com o aumento da idade.^31^^,^^40^

Diversos estudos também indicaram que a detecção, tratamento e controle da pressão arterial alta são fundamentais para reduzir a incidência de eventos cardiovasculares^41^ O estudo de Framingham apontou que a pressão elevada aumenta a chance de eventos cardiovasculares, sendo ainda mais elevada na ausência de tratamento.

O ERG aumenta com o tabagismo,^9^^,^^21^ o que já e altamente documentado na literatura, incluindo os estudos de Framingham.^20^^,^^22^ As diretrizes cardiovasculares recomendam fortemente a cessação do tabagismo como medida prioritária na prevenção secundária das doenças cardiovasculares e outras doenças crônicas não transmissíveis (DCNT).^21^

Indivíduos que se autopercebem com saúde ruim ou muito ruim mostraram RCV quase oito vezes mais elevado entre mulheres e três vezes entre homens. A autoavaliação de saúde constitui um excelente preditor de mortalidade e de eventos graves, tanto em estudos internacionais^42^ quanto em nacionais,^43^ devido à percepção de risco do indivíduo, em função de sintomas, mudanças implementadas no estilo de vida em função da doença, como maior frequência aos serviços de saúde, consultas médicas, uso de medicamentos e também limitação das atividades diárias.^43^

O estudo aponta maior ERG em indivíduos com baixa escolaridade, o que já foi identificado em outros estudos internacionais^44^ e nacionais como o ELSA.^45^ As adversidades socioeconômicas têm forte associação com morbimortalidade por doença cardiovascular (DCV),^46^ aterosclerose subclínica, manifestações piores, indicadores metabólicos,^47^ consequência das desvantagens socioeconômicas, adversidades na infância,^45^ pior acesso a serviços de saúde e práticas de promoção a saúde e prevenção.^48^ Nesse sentido, os resultados reforçam a importância de se levar consideração variáveis socioeconômicas no planejamento de políticas públicas de prevenção de DCV.

Dentre os limites do estudo, cita-se o emprego de algoritmos a partir do estudo de Framingham. Pelo fato de o estudo de Framingham ter sido realizado há várias décadas, pode ter ocorrido mudança nos riscos para DCV, além de não refletir necessariamente o que ocorre em outras populações em função de diferenças étnicas, culturais e outras.^22^ Outra limitação consiste na não inclusão no cálculo de outros fatores de risco como dieta, peso corpóreo, atividade física, condições clinicas e uso de medicação para controle de colesterol.^21^^,^^24^ Por tratar-se de um desenho transversal, não sendo possível acompanhar os desfechos futuros como nos estudos longitudinais. A base laboratorial utilizada apresentou perdas amostrais, que foram minimizadas pelas ponderações utilizadas. Entretanto, os vícios podem não ter sido corrigidos, estando as estimativas sujeitas a revisão em estudos futuros.

No Brasil, o estudo longitudinal de saúde do adulto (Elsa, Brasil), que usou calculadoras distintas, calculou o risco cardiovascular em 10 anos em 6,9% e 7,6%.^42^ Essas diferentes classificações apontam a necessidade de explorar em próximos estudos outras classificações de risco cardiovascular, incluindo nos escores outros fatores de risco como: obesidade abdominal, alimentação inadequada, inatividade física.^21^^,^^49^

As calculadoras de risco cardiovascular têm sido amplamente utilizadas para identificar populações em risco e que devam ser alvo de medidas de promoção, prevenção e tratamento. Os protocolos podem variar conforme o consenso dos especialistas, mas em todos eles se recomendam medidas de alimentação saudável, incluindo consumo de frutas e hortaliças, redução de sal, gordura e açúcar, cessar o tabagismo, incluindo abordagens de aconselhamento e/ou medicamentosa, conforme o caso, reduzir o uso de álcool, atividade física, abordagem para obesidade e excesso de pesos, tratamento não medicamentoso combinado com medicamentoso para pacientes com hipertensão, diabetes, colesterol elevado e outras alterações, dependendo das características específicas.^21^ Essas abordagens devem ser monitoradas, definindo-se o alvo terapêutico e monitorando-se a evolução.

## Conclusion

O estudo identifica o EGR em 10 anos na população adulta brasileira sendo estimado o risco de 8,7 % entre mulheres e 21,6% entre homens. Indivíduos com alto RCV requerem modificação mais agressiva nos fatores de risco.^21^ O EGR poderia ser usado ainda para monitorar os progressos de pacientes em tratamento e melhora em suas pontuações de risco. Esses dados apontam a necessidade de avançar em ações preventivas, principalmente dirigir estratégias populacionais em busca de populações de alto risco, que em geral incluem abordagens medicamentosas e não medicamentosas.
