# Supplementary material for: Estimativas do Risco Cardiovascular em Dez Anos na População Brasileira: Um Estudo de Base Populacional
Source: Arq Bras Cardiol. 2021 Mar 3;116(3):423–31. [Article in Portuguese] doi: 10.36660/abc.20190861 (PMC8159568; doi:10.36660/abc.20190861)
Supplement: Translation

# Translation: Cardiovascular Risk Estimates in Ten Years in the Brazilian Population, a Population-Based Study

**Authors:** Deborah Carvalho Malta, Pedro Cisalpino Pinheiro, Renato Azeredo Teixeira, Isis Eloah Machado, Filipe Malta dos Santos, Antônio Luiz Pinho Ribeiro

**Affiliations:** 1 Belo HorizonteMG Brazil Universidade Federal de Minas Gerais, Belo Horizonte, MG - Brazil.; 2 Ouro PretoMG Brazil Universidade Federal de Ouro Preto, Ouro Preto, MG - Brazil.

**Keywords:** Cardiovascular Diseases, Risk Factors, Cholesterol, Atherosclerosis, Diabetes Mellitus, Hypertension, Epidemiology

## Abstract

**Background:**

Cardiovascular diseases are the leading cause of morbidity and mortality, resulting in high health costs and significant economic losses. The Framingham score has been widely used to stratify the cardiovascular risk of the individuals, identifying those at higher risk for the implementation of prevention measures directed to this group.

**Objective:**

To estimate cardiovascular risk at 10 years in the adult Brazilian population.

**Methods:**

Cross-sectional study using laboratory data from a subsample of the National Health Survey. To calculate cardiovascular risk, the Framingham score stratified by sex was used.

**Results:**

Most women (58.4%) had low cardiovascular risk, 32.9% had medium risk and 8.7% had high risk. Among men, 36.5% had low cardiovascular risk, 41.9% had medium risk and 21.6% had high risk. The risk increased with age and was high in the low-educated population. The proportion of the components of the Framingham model, by risk and sex, shows that, among women at high risk, the indicators that mostly contributed to cardiovascular risk were: systolic blood pressure, total cholesterol, HDL, diabetes and tobacco. Among men, systolic blood pressure, total cholesterol, HDL, tobacco and diabetes.

**Conclusion:**

The study estimates, for the first time in Brazil, the risk of developing cardiovascular disease in ten years. The risk score is useful to support the prevention practices of these diseases, considering the clinical and epidemiological context.

## Introduction

Cardiovascular diseases (CVD) were responsible for approximately 17.9 million deaths in 2016, nearly 31% of the total deaths worldwide, constituting the most frequent cause of morbidity and mortality rates.^1^^–^^3^ Also in Brazil, in 2016, CVDs presented the highest mortality rates and disability-adjusted life years (DALYs), in both sexes.^4^^,^^5^ CVDs also stand out due to their high hospitalization and treatment costs in the Brazilian public health system (SUS, in Portuguese), in addition to the indirect costs caused by the reduction in productivity, medical leave from work, and the negative effects upon the quality of life of the affected individuals and their family members.^6^

The Framingham Heart Study (a cohort study), which began in 1948, was the first to identify the association between the main risk factors (RF) (hypertension, high cholesterol levels and smoking) and coronary disease.^7^ In the sequence of these findings, guidelines and protocols arose, which focused on a single RF, such as hypertension,^8^ or cholesterol,^9^ for the prevention of CVD. In 1993, studies from New Zealand were the first to use multiple risk factors in determining cardiovascular risk.^10^ Conducted by the Framingham team, the studies proposed a systematization by sex and age range, which predicted the risk of coronary disease development in the coming decade, considering the scores calculated using systolic blood pressure, total cholesterol, HDL cholesterol, diabetes, and smoking.^10^^,^^11^

The proposal of Framingham algorithms to predict CVD was incorporated into the Third Report of the Panel of Specialists in the detection, evaluation and treatment of high cholesterol (Adult Treatment Panel III), in 2001.^12^ What followed was the validation of these algorithms in black and white individuals in the United States,^13^^,^^14^ in various populations of Europe, the Mediterranean region, Asia, and throughout the world, with good outcomes.^15^^–^^19^

Other adaptations followed, most notably for the Overall Cardiovascular Risk, in 2008, proposed by the Framingham group,^20^ seeking to estimate the risk of cardiovascular events over a 10-year period, such as coronary artery disease (CAD), stroke, occlusive peripheral arterial disease (OPAD) or heart failure, over a 10-year period.^20^ This score has been frequently used worldwide and has also been used in Brazil, following Brazilian guidelines, to understand and estimate the absolute CV risk over a 10-year period.^21^ These scores allow for preventive actions, especially since they guide the population-based strategy to search for and identify high risk, seeking opportunities for their prevention.^22^

In an attempt to understand the health profile of the Brazilian population, the Ministry of Health and the Brazilian Institute of Geography and Statistics (IBGE) conducted the National Health Survey (NHS), a broad household survey that gathered information on a national scale about the population. This questionnaire included information about CVD risk factors. In 2014 and 2015, laboratory exams were collected to make advancements in cardiovascular risk (CVR) assessments representative of the Brazilian population, considering that previous estimations have been based on specific population studies, such as hospital studies^23^ or cohort studies among employees from Brazilian universities.^24^

Therefore, the present study sought to estimate CVR over a 10-year period in the Brazilian adult population, according to NHS laboratory data.

## Methods

This is a cross-sectional study conducted by means of secondary data from the NHS, a Brazilian household survey, as part of the Integrated System of Household Surveys (ISHS), from IBGE.^25^^,^^26^ The laboratory component was collected in 2014 and 2015, and the NHS sampling and the laboratory subsample methodologies can be found in previous studies.^25^^,^^27^^,^^28^ The laboratory subsample included 8,952 people and, taking into account the correction for possible biases in the statistical analyses, post-stratification weights were used, according to sex, age, level of education, and region.^28^, ^29^ The weighting procedure used variables from both the samples and the reference population, obtained from external sources, according to data from the 2010 IBGE Census, to adjust the distribution of the collected sample in the household survey to that found for the complete groups of the Brazilian population. The choice of variables used in the construction of weights took into consideration the characteristics of the excluded population to minimize the representation bias. In this sense, using the post-stratification weights, the laboratory sample becomes representative of the Brazilian adult population.^28^^,^^29^

The blood collected in the laboratory was centrifuged, and the serum and plasma samples were stored in a refrigerator at 4 ºC and analyzed by automated and regularly calibrated equipment. Among the collected exams, glycated hemoglobin (HbA1c) was collected in a tube with ethylenediaminetetraacetic acid (EDTA) and dosed by High Pressure Liquid Chromatography (HPLC). This study used the cutoff point established by the World Health Organization (WHO), while the American Diabetes Association recommended HbA1c≥6.5% for the diagnosis of diabetes mellitus (DM).^29^ Total cholesterol (TC) and high-density lipoprotein (HDL) were collected in a gel tube and the values for the Brazilian population were calculated.^30^

Blood pressure was measured after explanation of the procedure to the patient, who was supposed to rest for at least five minutes in a calm environment; not have a full stomach; not have practiced physical exercise for 60 to 90 minutes prior; not have ingested alcoholic beverages, coffee or food; not have smoked for 30 minutes prior; maintaining legs crossed, feet on the ground, back resting on a chair, relaxing and not speaking during the measurement.^31^ In total, three measurements were taken, with intervals of two minutes between each, using a calibrated mercury column sphygmomanometer. At the end, the three measurements were recorded as the definitive value for data analysis.

Smoking was evaluated through the following questions: “Are you or have you ever been a smoker, that is, have you smoked at least 100 cigarettes throughout your life?” and “How many cigarettes do you currently smoke per day?”

The scoring to estimate the overall CVR followed that proposed by Framingham^20^ and considered sex, age, TC and HDL cholesterol, treated and untreated blood pressure, smoking (yes or no), diabetes (yes or no). Separate calculations were performed for men and women. The specific risks were calculated by age and considered the frequency rates (FR) described below.^20^ Individuals younger than 30 and older than 74 excluded from the analysis, maintaining the same age groups of the cohort used in the risk estimation.^20^ Likewise, individuals who declared that they had been diagnosed by a doctor with heart disease or stroke (also known as a cerebrovascular accident – CVA) were excluded from this analysis.

The scores considered that proposed by D’Agostino et al.,^20^ detailed in another publication,^20^ and which was adopted in Brazil, in 2013, by the Brazilian Society of Cardiology, entitled the Global Risk Score (GRS).^21^ Age was self-reported by the participants and considered the following age ranges: 30–34, 35–39, 40–44, 45–49, 50–54, 55–59, 60–64, 65–69, 70–74, 75 and over. The male scores ranged from 0 to 15 points and the female scores ranged from 0 to 12 points.

Male smokers presented scores of 4 points, while female smokers presented scores of 3 points. Blood pressure (BP) attributed a differential score between those that were and those that were not undergoing drug treatment, considering the question: “Have you used any high blood pressure drugs in the last 15 days?”. The male score ranged from -2 to 3 (under treatment) and 0 to 4 (without treatment), and the female score ranged from -1 to 7 (under treatment) and -3 to 5 (without treatment).^20^

Regarding laboratory exams, the cutoff points and estimation scores were:

- Diabetes: hemoglobin was used (HbA1c<6.5% = 0 for both sexes; HbA1c≥6.5% men = 3 points, women = 4 points), or disease diagnosis by a doctor.
- CT: For women: CT<160 mg/dl = 0 points, CT 160–199 mg/dl = 1 point, CT≥200 -239 mg/dl = 3 points, CT≥240 -279 mg/dl = 4 points, CT≥280 = 5 points). For men: CT<160 mg/dl = 0 points, CT 160–199 mg/dl = 1 point, CT≥200 -239 mg/dl = 2 points, CT≥240 -279 mg/dl = 3 points, CT≥280 = 4 points).
- HDL cholesterol for men (≥60 mg/dL= -2 points, HDL 50–59 = -1 point, HDL 45–49= 0 points, 35–44= 1, <35 mg/dL = 2 points). For women: ≥60 mg/dL = -2 points, HDL 50–59 mg/dL = -1 point, HDL 45–49 = 0 point, 35–44= 1, <35 mg/dL = 2 points).

The study estimated the overall GRS for men and women and the respective confidence intervals (95% CI). The analyses were carried out using Stata, version 13. According to the guidelines set forth by the Brazilian Society of Cardiology, the following cutoff points for cardiovascular risk over a 10-year period were used: a) low CVR <5%, intermediate CVR (5 to <20%) and high CVR (≥20%).^21^^,^^32^

The NHS questionnaire and the variables have been published in prior publications and greater details can be found in other publications.^27^ According to that set forth in the study protocol, all of the results of the exams were informed to the user by the laboratory in charge. In cases of abnormal results, the users were advised to seek out medical assistance in public health services. In cases of extreme risk, the users were contacted directly by the partner laboratory or by the Ministry of Health, attempting to provide immediate medical care assistance.^28^

It should also be noted that the NHS was approved by the National Ethics Commission on Research, logged under protocol number 328.159, on June 26, 2013. All individuals were consulted, their doubts clarified, and agreed to participate in this study.

## Results

This study shows that 58.4% of women presented low cardiovascular risk (<5%); 32.9% intermediate GRS (5 to 19%) and 8.7% high GRS (>=20%). The high GRS in women increased with age, from 0.1% in the 40–44-year-old group to 9.3% in the 50–54-year-old group; 10.6% in the 55–59-year-old group; 29% in the 60–64-year-old-group; 29.9% in the 65–69-year-old group, and 38.4% in the 70–74-year-old group. The difference in the GRS according to years of education was nearly five-fold comparing the high level of education (12 years or more) with the low level of education (<8 years) (3.2%: 95% CI 2.4–4.4 versus 15.7%: 95% CI 13.5–18.3). Those who had health insurance presented a lower GR, 5.4% (95% CI 3.9–7.3) versus 10.2% (95% CI 8.8–11.8) of those who did not. Black women represented the largest proportion in the high-risk group (>=20%): 14.4% (95% CI 9.7–20.9), compared to white women, 7.3 (95% CI 5.8–9.1). The self-evaluation of bad health showed the largest difference among women, and presented a gradient, considering the following extremes: women who self-evaluate themselves as having good health, 2.9% (95% CI 1.3–3.6), and very bad health, 25.6% (12.7–45.0) (Table 1).

**Table 1:** Proportional distribution of the selected variable by cardiovascular risk groups, women, NHS 2013

Variable		Less than 10%		Between 11% and 20%		Greater than 20%	
		n	%	n	%	n	%
**Women**		2092	58.4 (56.3;60.5)	1180	32.9 (31;35)	312	8.7 (7.6;9.9)
**Age**							
	30–34	564	100	0	-	0	-
	35–39	482	94.1 (90.2;96.5)	30	5.8 (3.4;9.8)	0	0 (0;0.3)
	40–44	404	84.9 (80.2;88.6)	72	15 (11.3;19.7)	1	0.1 (0;0.9)
	45–49	353	70.3 (64.8;75.3)	147	29.3 (24.3;34.8)	2	0.4 (0.1;1.6)
	50–54	161	39.2 (33.3;45.5)	211	51.4 (45.2;57.6)	38	9.3 (6.3;13.7)
	55–59	87	22.1 (17.1;28)	265	67.3 (61;73)	42	10.6 (7.6;14.7)
	60–64	28	9.3 (6.2;13.6)	186	61.7 (54.8;68.2)	87	29 (23;35.9)
	65–69	12	4.5 (2.4;8.5)	172	65.5 (58.2;72.2)	79	29.9 (23.6;37.2)
	70–74	2	1.5 (0.5;4.1)	98	60.2 (50.5;69.1)	63	38.4 (29.5;48.1)
**Level of education**							
	0–8 years	572	40.2 (37.1;43.3)	628	44.1 (41;47.2)	224	15.7 (13.5;18.3)
	9 to 11	293	61.5 (55.3;67.3)	151	31.6 (26.1;37.7)	33	6.9 (4.5;10.4)
	12 and over	1227	72.9 (70;75.7)	401	23.8 (21.2;26.7)	54	3.2 (2.4;4.4)
**Skin color**							
	White	1003	58.3 (55;61.6)	591	34.4 (31.3;37.6)	125	7.3 (5.8;9.1)
	Black	169	49.8 (42.7;56.9)	122	35.8 (29.4;42.7)	49	14.4 (9.7;20.9)
	Light-skinned Black	891	60.1 (57.2;62.8)	457	30.8 (28.3;33.5)	135	9.1 (7.6;10.9)
	Other	30	69.4 (49.1;84.2)	11	24.8 (11.4;45.7)	2	5.9 (2.2;14.8)
**Region**							
	North	150	61.8 (58.5;65)	63	29.5 (26.5;32.6)	12	8.7 (7;10.8)
	Northeast	561	54.8 (52.1;57.4)	278	32.7 (30.3;35.2)	85	12.6 (11;14.4)
	Southeast	910	49.9 (46.2;53.5)	578	36.3 (32.9;39.9)	152	13.8 (11.6;16.4)
	South	308	50.7 (46.3;55)	177	36.1 (32.1;40.2)	46	13.3 (10.7;16.3)
	Midwest	164	54.9 (50.3;59.5)	84	33.1 (28.9;37.5)	16	12 (9.4;15.2)
**Health Insurance**							0 (0;0)
	No	1375	56 (53.5;58.4)	830	33.8 (31.5;36.2)	251	10.2 (8.8;11.8)
	Yes	717	63.6 (59.6;67.4)	350	31.1 (27.4;35)	60	5.4 (3.9;7.3)
**Self-evaluation**							
	Very good	313	73.4 (67;78.9)	102	23.8 (18.5;30)	12	2.9 (1.3;6)
	Good	1188	67.3 (64.3;70.1)	500	28.3 (25,6;31.2)	78	4.4 (3.3;5.8)
	Regular	504	44.3 (40.9;47.8)	469	41.2 (37.7;44.8)	165	14.5 (12.1;17.2)
	Bad	69	33.1 (26;41.2)	94	45.1 (37.4;53.1)	45	21.7 (15.9;29)
	Very bad	17	39.2 (24.5;56.2)	16	35.1 (22.6;50.2)	11	25.6 (12.7;45)

Source: National Health Survey, 2013.

Among men, 36.5% presented low cardiovascular risk (<5%); 41.9% presented intermediate GRS (5 to 19%); and 21.6%, high GRS (> 20%). The high GRS in men increased with age, from 1.0% in the 40–44-year-old group; 4.9% in the 45–49-year-old group; 17.1% in the 50–54-year-old group, 44.7% in the 55–59-year-old group; 61.5% in the 60–64-year-old group; 78.2% in the 65–69-year-old group, 91.9% in the 70–74-year-old and older group. The difference in the CVR, according to the level of education, was nearly twice as high, 13.8% (12 years or more) and 29.8% (<8 years). No difference was identified in the GRS considering race and skin color, and having health insurance. The self-evaluation of health in men also presented a gradient: very good, 11.4% (95% CI 8–15.9) and the self-evaluation of bad health, 39.1% (95% CI 28.8–50.4) (Table 2).

**Table 2:** Proportional distribution of selected variables by cardiovascular risk groups, men, NHS 2013

Variable		Less than 10%		Between 11% and 20%		Greater than 20%	
		n	%	n	%	n	%
**Men**		950	36.5 (34.1;39.1)	1088	41.9 (39.4;44.4)	562	21.6 (19.7;23.6)
**Age**							
	30–34	390	96.4 (92.9;98.2)	15	3.6 (1.8;7.1)	0	–
	35–39	342	84,5 (78.9;88.7)	63	15.5 (11.3;21.1)	0	–
	40–44	139	38,2 (31.8;45)	221	60.8 (53.9;67.2)	4	1 (0.4;2.4)
	45–49	63	18.7 (13.9;24.6)	258	76.4 (70.2;81.7)	16	4.9 (2.7;8.5)
	50–54	17	5.1 (3;8.6)	250	77.7 (71.7;82.7)	55	17.1 (12.7;22.8)
	55–59	0	–	142	55.3 (47.4;62.9)	115	44.7 (37.1;52.6)
	60–64	0	–	99	38.5 (31.3;46.2)	158	61.5 (53.8;68.7)
	65–69	0	–	33	21.8 (15.7;29.4)	118	78.2 (70.6;84.3)
	70–74	0	–	8	8.1 (4.2;15.2)	95	91.9 (84.8;95.8)
**Level of education**							
	0–8 years	237	21.8 (19.1;24.8)	525	48.4 (44.8;51.9)	324	29.8 (26.8;33.1)
	9 to 11	163	41.8 (35.1;48.8)	145	37.1 (30.7;44)	82	21.1 (16.2;27)
	12 and over	550	48.9 (44.8;53.1)	418	37.2 (33.3;41.3)	155	13.8 (11.4;16.7)
**Skin color**							
	White	408	33.5 (29.8;37.5)	532	43.7 (39.8;47.7)	277	22.8 (19.8;26.1)
	Black	92	34 (26.8;42.1)	129	47.9 (39.6;56.3)	49	18.1 (12.7;25)
	Light–Skinned black	441	40.5 (36.8;44.2)	415	38.2 (34.8;41.6)	233	21.4 (18.8;24.3)
	Other	10	41.1 (23.1;61.9)	12	47.4 (27;68.7)	3	11.5 (4.9;24.7)
**Region**							
	North	70	39.7 (35.7;43.7)	78	44.3 (40.3;48.3)	28	16.1 (13.4;19.2)
	Northeast	274	40.2 (36.9;43.6)	283	41.6 (38.4;45)	124	18.2 (15.7;20.9)
	Southeast	391	34.2 (29.6;39.2)	463	40.5 (35.8;45.4)	288	25.2 (21.6;29.3)
	South	143	3.,9 (30.3;42)	170	42.9 (37.2;48.8)	84	21.2 (17.1;25.9)
	Midwest	72	35.8 (30;41.9)	93	45.9 (40.1;51.8)	37	18.3 (14.4;23)
**Health insurance**							
	No	649	34.9 (32.1;37.8)	790	42.6 (39.7;45.4)	418	22.5 (20.3;24.9)
	Yes	302	40.6 (35.5;45.9)	298	40.1 (35.1;45.3)	143	19.3 (15.7;23.5)
**Self–evaluation**							
	Very good	210	52.8 (45.7;59.8)	143	35.8 (29.5;42.7)	45	11.4 (8;15.9)
	Good	566	41 (37.5;44.6)	562	40.7 (37.3;44.2)	254	18.4 (15.9;21.1)
	Regular	159	22.7 (19.2;26.7)	323	46.2 (41.6;50.8)	218	31.1 (27.1;35.4)
	Bad	13	13.3 (8.4;20.5)	46	47.6 (36.3;59.2)	37	39.1 (28.8;50.4)
	Very bad	2	7.6 (2.2;22.7)	14	61.3 (39.1;79.7)	7	31.1 (14.7;54.2)

Source: National Health Survey, 2013.

Figure 1 shows the proportional distribution of the Framingham model components, by risk groups, which contributed positively (greater than zero) to the total score. In the high risk among women, the indicators that most contributed to the GRS were: systolic blood pressure (97.7%), TC (91.3%), diabetes (62.8%), HDL cholesterol (60.6%) and smoking. In the high risk among men, the indicators that most contributed to the GRS were: TC (85%), systolic blood pressure (84.3%), HDL cholesterol (76.2%), smoking (39.9%) and diabetes (24.7%).

**Figure 1:** Proportional distribution of the components of the Framingham model by high, intermediate, and low-risk groups, respectively, by sex, NHS 2013.

Source: National Health Survey, 2013

## Discussion

This study is the first national population-based study to estimate the GRS for the Brazilian adult population using laboratory data. For the calculation, algorithms were employed by D’Agostino et al.^20^ according to findings from the Framingham study. These models were estimated by mathematic functions to estimate the absolute risk of CVD in a 10-year period.^20^ A high GRS (>=20%) was found in nearly 8.7% of the women and nearly one fifth of the men. GRS increased with age, affecting approximately 40% of the women between 70 and 74 years of age and nearly all of the men in this age range. The risk quadrupled among women with low level of education and doubled among men. It is worth noting that there is a large concentration of individuals with GRS greater than 20% in the groups with low levels of education and older individuals. Part of this concentration may be an effect from the cohort, given that, on average, older individuals are less educated than the younger ones.^33^ An important portion of the concentration of the less educated individuals in the high-risk group can be explained by the more advanced age of the group and vice-versa. Other analyses, which are not in the scope of this study, can separate the effects.

Only black women, compared to white women, represented a larger proportion in the high-risk group. What is surprising is the absence of a statistically significant race/color difference in the percentage of men with high GRS. It is likely that part of the differential potential by race has been captured by other correlated variables, such as age and level of education. In the sample, the white male population, as compared to the blacks and light-skinned blacks, show a greater concentration in older ages. Among women, the risk was higher among those that do not have health insurance and a dose-response gradient was also observed between CVR and the self-evaluation of health, reaching eight-fold higher levels between the very good and very bad evaluations, whereas among men this difference was approximately 3-fold higher. The factors that most frequently contributed to the high GRS were age, blood pressure and high cholesterol.

Various risk assessment calculations were developed to estimate the CVR according to the Framingham study findings. The current score was revised in 2008^20^ and includes additional cardiovascular clinical parameters. Although this risk model provides an improved CVD estimate, it still faces some challenges and underestimates the risk in women.^21^ The classification employed in this study used the Cox model and covariables, such as age, TC, HDL cholesterol, treated and untreated systolic blood pressure, antihypertensive medications, current smoking and the status of diabetes with the CVR calculation.^20^ The authors transformed the continuous variables into logarithms to improve the discrimination and calibration of the models and to minimize the influence of extreme observations.^20^

The algorithms were recommended by the Brazilian Society of Cardiology in the first version of the Brazilian Guidelines for Cardiovascular Disease Prevention^21^ and add advantages in the identification of the GRS, selecting priority individuals for intervention, with multiple risk factors, avoiding the unnecessary identification of people with only one isolated risk.^20^ These estimations of global CVD support the identification of selected patients for prevention and treatment measures, making the measures cost-effective^21^ and useful for application in primary care.

The proposed algorithm is classified according to sex, increasing the risk score with increasing age, smoking habits, untreated BP and diabetes.^20^ Among women, the algorithm increases in post-menopause age ranges and raises the risk for such factors as smoking and diabetes. Despite the use of higher scores for women, the GRS was still twice as high among men.

In Brazil, some studies measured the CVR among adults and the elderly, employing the Framingham calculation,^34^ as seen in the Bambuí cohort. Adults (n=547, 30–59 years of age) and the entire elderly population (n=1165, 60–74 years of age) were analyzed, and the CVR among the elderly was found in 56% of the men and 21% of the women.^35^ Another national study, which evaluated approximately 15,000 individuals who received medical care in the check-up service of the Preventive Medical Center of the Israelita Albert Einstein Hospital between 2009 and 2015, also identified similar proportions. High CVR in women was 12.3% and in men was 40.1%.^23^

The higher CVR in men reflects the presence of less healthy lifestyles, such as smoking, improper diet, alcohol consumption, infrequent search for health services, non-use of medications, which has been documented in a number of other national studies.^35^^–^^37^

The increase in risk with age has been attributed to aging, increase in BP, which can affect 60% of the elderly, according to data from the NHS.^31^ The explanations would be the inherent changes that come with aging, including hardening of the arteries, greater peripheral vascular resistance and comorbidities among the elderly.^21^^,^^38^^–^^40^

In the case of women, the rise in GRS in the post-menopause age range results from the loss of the hormone prevention effect in this stage of life. The increase in hypertension in women has been described by the growth of central obesity with the increase in age.^31^^,^^40^

A wide range of studies have also indicated that detection, treatment and control of high blood pressure are crucial to reduce the incidence of cardiovascular events.^41^ The Framingham study pointed out that high blood pressure increases the chance of cardiovascular events, which is even higher in the absence of treatment.

The GRS increases with smoking,^9^^,^^21^ which is highly documented in the literature, including in the Framingham studies.^20^^,^^22^ The cardiovascular guidelines highly recommend that the patient stop smoking as a priority measure in the secondary prevention of cardiovascular diseases and other untreated cardiovascular diseases.^21^

Individuals who self-perceive their own health as bad or very bad presented CVR almost eight times higher among women and three times higher among men. The self-evaluation of health constitutes an excellent predictor of mortality and severe events, both in international^42^ and in national events.^43^ This is due to the individual’s own risk perception, brought about by the symptoms, lifestyle changes due to the disease, greater frequency of visits to healthcare services, doctor’s appointments, use of medications and the limitation of daily activities.^43^

This study highlights a greater GRS in individuals with low level of education, which has been identified in other international^44^ and national studies, such as ELSA-Brasil.^45^ The socioeconomic adversities have a strong association with morbidity and mortality through CVD,^46^ subclinical atherosclerosis, worse manifestations such as metabolic indicators^47^ a consequence of socioeconomic disadvantages, adversities in childhood,^45^ worse access to healthcare services, and health promotion and prevention practices.^48^ In this sense, the results reinforce the importance of taking into consideration socioeconomic variables in the planning of public policies for CVD prevention.

The limitations of this study include the use of algorithms from the study conducted by Framingham. Since Framingham’s studies were conducted many decades ago, CVD risks may have changed, and the study findings do not necessarily reflect what occurs in other populations as regards ethnic and cultural differences and others.^22^ Another limitation consists of the non-inclusion of other risk factors in the calculation, such as diet, body weight and physical exercise; clinical conditions; and the use of medication to control cholesterol.^21^^,^^24^ As this is a cross-sectional design, it was also impossible to follow up on future outcomes, as it occurs in longitudinal studies. The laboratorial base used in this study presented sample losses, which were minimized by the weighting used; however, the bias may not have been corrected, thus making the estimations subject to review in future studies.

In Brazil, the longitudinal study of adult health (ELSA-Brasil), using different calculations, calculated CVR over a 10–year period in 6.9% and 7.6%.^42^ These different classifications highlight the need, in future studies, to explore other CVR classifications, including in the scores of other risk factors, such as: abdominal obesity, improper diet, and a lack of physical activity.^21^^,^^49^

The CVR calculations have been widely used to identify at-risk populations and those that should be the target of health promotion, prevention and treatment measures. The protocols can vary according to the consensus of the specialists, but in all of these, a healthy diet is recommended, including the consumption of fruits and vegetables; reduction of salt, fat and sugar; stopping smoking, doctor’s advice or medical treatment, as needed; reduction in alcohol consumption; physical exercise; approaches toward obesity and overweight; non-drug treatments combined with medications for patients with hypertension, diabetes, high cholesterol; and other changes, depending on specific characteristics.^21^ These approaches should be monitored, defining the therapeutic target and monitoring the evolution.

## Conclusion

This study identified the GRS over a 10–year period in the Brazilian adult population, with an estimated risk of 8.7% among women and 21.6% among men. Individuals with a high CVR require more aggressive changes in their risk factors.21 The GRS can still be used to monitor the progress of patients in treatment and improve their risk scores. These data highlight the need for advances in preventive actions, primarily guiding population strategies in the search for high-risk populations, which, in general, include medication and non-medication approaches.
